# Complementary and alternative medicine - practice, attitudes, and knowledge among healthcare professionals in New Zealand: an integrative review

**DOI:** 10.1186/s12906-021-03235-z

**Published:** 2021-02-13

**Authors:** Lizhou Liu, Yong Tang, G. David Baxter, Haiyan Yin, Steve Tumilty

**Affiliations:** 1 Centre for Health, Activity, and Rehabilitation Research, School of Physiotherapy, University of Otago, PO Box 56, Dunedin, 9054 New Zealand; 2 China-New Zealand Collaboration Centre for Integrative Medicine (CHINZIM), University of Otago, Dunedin, New Zealand; 3 Acupuncture & Tuina School, Chengdu University of Traditional Chinese Medicine, Chengdu, China; 4 China-New Zealand Collaboration Centre for Integrative Medicine (CHINZIM), Chengdu University of Traditional Chinese Medicine, Chengdu, China; 5 Acupuncture & Chronobiology Key Laboratory of Sichuan Province, Chengdu, China

**Keywords:** Complementary and alternative medicine, New Zealand, Healthcare practitioners, Practice, Attitudes, Knowledge

## Abstract

**Background:**

The prevalence of CAM use is increasing. This integrative review investigated New Zealand healthcare professionals’ practice of, attitudes toward, and knowledge about complementary and alternative medicine (CAM).

**Methods:**

Literature search was conducted in four databases from inception to April 2020. Studies were included if they reported results from primary data collection on practice of, attitudes toward, or knowledge about CAM amongst New Zealand healthcare professionals.

**Results:**

Eleven studies (two of ‘high-quality’, seven of ‘moderate-quality’, and two of ‘low-quality’) were identified with 2060 healthcare professionals including general practitioners (GPs), nurses, midwives, pharmacists, physiotherapists, and medical specialists. New Zealand healthcare professionals were generally positive regarding CAM use, but have concerns on the scientific evidence, regulation, safety, financial costs of CAM, and encourage an evidence-based CAM practice and stronger CAM regulation. Findings indicated that around 25% of GPs practise CAM, and 82.3% refer patients to CAM practitioners. When treating pregnant women, 48.4% of physiotherapists practise acupuncture, and 37.3% of midwives recommend CAM. GPs believe that acupuncture is the most helpful CAM modality, and most commonly practiced and referred patients to acupuncture. Up to 58% of GPs and Plunket nurses wanted to receive further education on CAM, and up to 66.7% GPs favour the idea CAM should be included in medical curriculums.

**Conclusions:**

Nine of the 11 included studies were of moderate to high quality, thus enhancing the reliability of the review findings. In order to better manage CAM in New Zealand New Zealand clinical settings, there is a need to invest in CAM research and education, and enhance CAM regulation. This review is a first step in developing an evidence base to offer insights for further development of effective CAM policies regarding safety, efficacy, regulation and integration in New Zealand.

**Supplementary Information:**

The online version contains supplementary material available at 10.1186/s12906-021-03235-z.

## Background

Complementary and alternative medicine (CAM) is an umbrella term describing a range of health systems, modalities, and practices that are not generally considered part of conventional medicine. CAM modalities are divided into five main categories by the American National Center for Complementary and Integrative Health: alternative medical systems, biologically based treatments, manipulative and body-based methods, mind-body interventions, and energy therapies [1]. In New Zealand, it is estimated that there are 69 CAM modalities available [2].

Similar to other developed countries, public interest in and use of CAM in New Zealand has increased over the last decades, although CAM research is limited in this country. It has previously been reported by three regional surveys (2003–2006) that CAM therapies was used by 67% of patients at medical practices [3], 38% of emergency department presenters [4], and 49% of cancer patients [5]. The most recent national health survey (2006–2007) found that nearly one in five adults visited a CAM practitioner in the previous 12 months, with the number of women being significantly higher than men [6]. The types of CAM used were most commonly seeing a massage therapist, followed by a homoeopath or naturopath, and an acupuncturist (visits of chiropractor and osteopath were separately evaluated in this survey) [6].

Recognizing the potential impact of CAM on patient care, CAM is slowly gaining political recognition in New Zealand. The *Ministerial Advisory Committee on Complementary and Alternative Health (MACCAH)* was established in June 2001, in direct response to a request by the *Ministry of Health*. The committee finished its term in June 2004, providing information and advice to the Minister on complementary and alternative healthcare focusing on areas of regulation, consumer information needs, research, and integration [7]. In addition, at the national level, the Accident Compensation Corporation (ACC) provides compulsory no-fault personal injury cover for treatment for all New Zealand residents and visitors to New Zealand, which includes cover for acupuncture, chiropractic and osteopathy. This funding stream results in increasing numbers of patients who use CAM therapies in conjunction with mainstream medicine, and in turn the development of closer ties between CAM practitioners and a range of conventional healthcare professions in New Zealand. While the focus for evidence in CAM has been increasingly recognized at a national level, in 2018, the ACC undertook a review to synthesize the published academic literature on the effectiveness and safety of acupuncture interventions for the treatment of musculoskeletal conditions and injuries [8], indicating the emerging research on CAM in New Zealand.

Healthcare professionals’ attitudes toward and knowledge about CAM are important for patient care decision making, as they may affects the option of a multidisciplinary healthcare approach [9]. In addition, healthcare professionals’ beliefs and practice behaviours of CAM can significantly impact the development of CAM professions and their role in the healthcare system, despite studies that demonstrate the public’s desire for the integration of certain CAM modalities into the mainstream health system [8]. A literature review summarizing 21 surveys of physicians, nurses, public health professionals, dieticians, social workers, medical and nursing faculty, and pharmacists in Canada and United States, concluded that while physicians are more negative than other healthcare professionals, positive attitudes toward CAM are not related to CAM referral or prescription patterns [10]. It is unknown whether these findings are representative of healthcare professionals in other countries. Furthermore, as this review was published over ten years ago, the relevance of the reported findings may not be contemporary.

In New Zealand, the regulated health professional workforce numbered 97,786 in 2015 [11]. A number of localized surveys on specific practitioners’ use of and attitudes towards CAM (e.g., general practitioners [GPs]) have been conducted in New Zealand over the past 25 years [3, 8, 12–15]. There has yet to be a study evaluating the overall practice of, attitudes toward, and knowledge about CAM therapies among various categories of New Zealand healthcare professionals. This gap in the literature is particularly important given that conventional healthcare professionals are often reported to be reluctant or lacking in confidence to advise patients on the use of CAM, and therefore issues such as safety, effectiveness, and practicality of CAM use may be overlooked [9]. It will be useful to know their perceptions, knowledge and clinical behaviours related to CAM therapies and practitioners in order to inform further development of evidence-based CAM in New Zealand, e.g. in terms of the methodological design of clinical research, as well as the development of education packages for New Zealand healthcare professionals. This information will be of importance to further CAM development in New Zealand, in order to develop effective policies based on effectiveness, safety, regulation and integration [8].

The primary aim of this review was to summarize the results of existing studies (both quantitative and qualitative) that investigated New Zealand healthcare professionals’ practice of, attitudes towards, and knowledge about CAM. In addition, the secondary aim was to explore whether the CAM practice, attitudes, and knowledge are different amongst healthcare professionals or change over time. For these purposes, an integrative review approach was adopted, which allows for the combination of diverse methodologies to capture the context, processes, and subject elements of this topic [16].

## Methods

The methods for this review were consistent with the methodology framework for integrative reviews [16]. The protocol of this review was not registered. This review was reported in accordance to the *Preferred Reporting Items for Systematic Reviews and Meta-Analyses (PRISMA) guidelines* [17].

### Eligibility criteria

Studies were included if they met the following criteria:

1. Population: New Zealand healthcare professionals regulated under the *Health Practitioners Competence Assurance (HPCA) Act 2003* [18].
2. Outcome measures: practice, use, recommendation, attitudes, views, and knowledge of CAM.
3. Study type: both quantitative and qualitative studies that reported results on original empirical research findings. Reviews, study protocols, correspondence, commentaries, and letters were excluded.

### Search strategy

A comprehensive literature search was conducted by two independent researchers (LL and YT) in AMED, CINAHL, EMBASE, MEDLINE from inception to April 2020. The search strategy was developed following consultation with a health sciences librarian (Table 1). The four themes were combined with “AND”, while the individual terms in each theme combined with “OR”. The MEDLINE strategy was first finalized and then adapted for search in other databases. There were no limitations on language or year of publication. Reference lists of included studies and relevant reviews were manually searched for additional references.

**Table 1:** Summary of the search terms

THEME 1: CAM terms		THEME 2: Outcomes	THEME 3: Healthcare professional terms	THEME 4: Limiters
***Broad descriptor headings***	***Specific headings***			
alternative medicine/therapy	acupuncture	attitude*	consultant*	Aotearoa
CAM	aromatherapy	belief*	dental*	Māori
complementary and alternative medicine	chiropractic	knowledge*	dentist*	New Zealand
complementary medicine/therapy	dietary supplements	perception*	dietitian*	
integrative medicine/therapy	herb* medicine	perspective*	doctor*	
traditional medicine/therapy	homeopathy	practice*	general practitioner*	
	hypno*	recommendation*	health* professional*	
	massage	use*	midwi*	
	meditation	value*	nurse*	
	naturopathy	view*	occupational therapist*	
	osteopathy		oral health therapists*	
	reflexology		paramedic*	
	rongoā		pharmacist*	
	spiritual healing		physiotherapist*	
	traditional Chinese medicine		podiatrist*	
	traditional Māori medicine		psychologist*	
	traditional Pacific Island medicine		psychotherapist*	
	vitamins		optometrist*	
	yoga			

### Selection of studies

Titles, abstracts, and full text of potential articles were reviewed by two independent reviewers (LL and YT) for eligibility. Consensus was reached by discussion, and a third reviewer (ST) involved for the final decision where disagreement persisted.

### Data extraction

Data were extracted from the included studies by the first author (LL), and separately reviewed by a second reviewer (YT). Findings were compared and agreed upon discussion. Further data were obtained through email contacts with corresponding authors if necessary. The window for response was set at four weeks; a follow up email was sent to non-responders after three weeks. Required information included general information (first author and year of publication), study characteristics (study design, sampling method, sample size, response rate, and the investigated CAM therapies), participant characteristics (target population, age, ethnicity, and gender), and three key themes of investigation emerged from included studies (‘prevalence of practice, use and referral’, ‘attitudes and views’, ‘knowledge and education).

### Quality assessment

Methodological quality of included studies was assessed by two independent reviewers (LL and YT) with disagreement resolved through consensus method. The quality of quantitative studies was assessed using the Hoy’s risk of bias tool for observational studies [19]. This 10-item checklist addresses four domains of bias (selection bias, nonresponse bias, measurement bias, and bias related to the analysis) plus a summary risk of bias assessment. The summary assessment evaluates the overall risk of study bias (low, moderate, high) which is based on the reviewer’s judgment given responses to the preceding ten items. The quality of qualitative studies was assessed using the *Critical Appraisal Skills Programme (CASP) (2018) for qualitative research* [20]. The *CASP checklist* assesses 10 questions that are considered important for appraising the quality of qualitative research: aim, methodology, design, recruitment strategy, data collection, relationship between researcher and participants, ethical issues, data analysis, findings statement, and research value. No formal rating scale for this checklist is available; therefore, for the purpose of this review, a rating system previously used in other reviews was employed: a study was considered as ‘high’ quality if the total score was over 6, 4–6 as ‘moderate’ quality, and less than 4 as ‘low’ quality [21, 22].

### Data synthesis

The data from included studies were grouped and summarized in a narrative manner in accordance with the three key themes. CAM practice, attitudes and knowledge among different categories of healthcare professionals were compared where data permitted. Changes in CAM practice, attitudes and knowledge over time were summarized by professional categories.

## Results

### Study selection

**Fig. 1:**
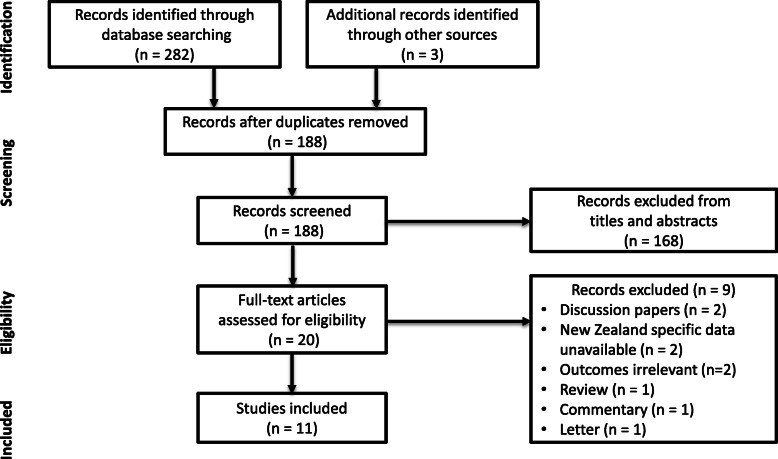
Flow diagram of study selection

A total of 285 articles were identified using the search criteria. After removing 97 duplicates, 168 were excluded based on titles and abstracts, and 20 screened for full text for eligibility. Eleven articles met the selection criteria, and were included in this review. Of the nine excluded articles, two were discussion papers [23, 24], two reported international studies without data specific to New Zealand [25, 26], two reported outcomes irrelevant to this review [27, 28], and the remaining three were a review [29], a commentary [30], and a letter [31]. Figure 1 displays the process of study selection.

### Study characteristics

Characteristics of the 11 included studies are presented in Table 2. A total of 2060 healthcare professionals were included, with a range of 5 to 500 participants per study. The investigated healthcare professionals included GPs (*n* = 7 studies) [3, 8, 12–15, 33], Plunket nurses (registered child health nurses working in the community) (*n* = 1) [9], midwives (n = 1) [32], pharmacists (n = 1) [34], physiotherapists (n = 1) [35], and specialists (n = 1) [15]. The CAM modalities investigated in the studies varied greatly: one study [35] focused solely on acupuncture, one [8] (mainly) on massage, two [12, 14] on a variety of CAM therapies from the list selected from the NCCAM, including acupuncture, aromatherapy, chiropractic, herbal medicines, homeopathy, hypnosis, naturopathy, osteopathy, reflexology, spiritual healing, traditional Chinese Medicine, traditional Māori Medicine, traditional Pacific Island Medicine; the remaining seven [3, 9, 13, 15, 32–34] did not specify the definition of CAM. Most participants were middle aged, New Zealand European. Of the 11 included studies, there were eight surveys [3, 8, 12–15, 32, 35, 36] and three qualitative studies (two interviews [33, 34] and one focus group study [9]).The sampling methods used included census (*n* = 4) [3, 12, 15, 35], convenience (*n* = 1) [9], simple random (*n* = 3) [8, 13, 14], systematic random (*n* = 1) [32], stratified (n = 1) [33], and a mixed use of purposive and convenience approaches (n = 1) [34]. Instruments used in the eight surveys varied, without psychometric properties reported; four studies used instruments that were developed and adapted from previous studies [8, 14, 15, 35]. Response rates for surveys ranged from 43% [8] to 83.3% [3]. Two studies were of ‘high’ quality [9, 34], seven of ‘moderate’ quality [3, 8, 12, 14, 32, 33, 35], and two of ‘low’ quality [13, 15] according to the Hoy’s critical appraisal tool and CASP checklist (see Additional files 1 and 2).

**Table 2:** Characteristics of the 11 studies included in the review

Author (Year)	Method	Target population	Investigated CAM therapies	Sample size/RR^c^	Sampling method	Main age group (%)	Ethnicity (NZ European %)	Male (%)	Theme^d^			Study quality
									I	II	III	
Hadley (1988) [12]	Survey	GPs	Seven CAM therapies^a^	226/77%	Census	30-50 yr (58%)	NR	75	√	×	√	Moderate
Marshall (1990) [13]	Survey	GPs	NS	370/67.3%	Simple random	Mean = 44 yr	84.7	82.6	√	√	√	Low
Taylor (2003) [3]	Survey	GPs	NS	30/83.3%	Census	NR	NR	NR	√	√	√	Moderate
Lawler (2004) [8]	Survey	GPs	Massage and other CAM	200/43%	Simple random	Mean = 46.1 yr	73	57	√	√	√	Moderate
Poynton (2006) [14]	Survey	GPs	Thirteen CAM therapies^b^	500/60%	Simple random	Mean = 50.3 yr	72	60	√	×	√	Moderate
Harding (2009) [32]	Survey	Midwives	NS	171/44.6%	Systematic random	NR	NR	NR	√	√	×	Moderate
Bocock (2011) [15]	Survey	GPs & specialists	NS	395/59%	Census	41-50 yr (36%)	88	66	√	√	√	Low
Upsdell (2011) [33]	Interview	GPs	NS	12	Stratified	NR	NR	NR	×	√	√	Moderate
Lo (2012) [9]	Focus group	Plunket nurses	NS	5	Convenience	20-40 yr (100%)	60	0	×	√	√	High
Barnes (2018) [34]	Interview	Pharmacists	NS	27	Purposive & convenience	40-49 yr (44.4%)	NR	51.9	×	√	√	High
McDowell (2019) [35]	Survey	Physios	Acupuncture	124/NR	Census	30-40 yr (38.7%)	NR	21	√	√	√	Moderate

*Abbreviations*: *NS* not specified, *GPs* general practitioners, *CAM* complementary and alternative medicine, *NZ* New Zealand, *physios* physiotherapists, *RR *response rate

^a^ The specified seven therapies were hypnosis, acupuncture, osteopathy, chiropractic, naturopathy, homeopathy and spiritual healing

^b^ The specified thirteen therapies were acupuncture, aromatherapy, chiropractic, herbal medicines, homeopathy, hypnosis, naturopathy, osteopathy, reflexology, spiritual healing, traditional Chinese Medicine, traditional Māori Medicine, traditional Pacific Island Medicine

^c^ RR applied to survey studies only

^d^ I = prevalence of practice, use and referral for CAM; II = attitudes and views toward CAM, III = knowledge and education regarding CAM

### Prevalence of practice, use and referral for CAM

The practice, self-use, and referral for patients to CAM therapies by healthcare professionals appears common in a New Zealand clinical setting. The prevalence of CAM practice ranged from 20.3% [14] to 30.1% [13] among GPs, and 48.4% among physiotherapists when treating women during pregnancy [35]. It was reported that 47% of GPs personally use massage [8], and approximately 26% of GPs and specialists use CAM to treat their own ailments [15]. The proportion of professionals reporting CAM referrals ranged from 37.3% [32] to 94.7% [14].

Reported reasons for using CAM therapies included failure of conventional treatment, patient request, past positive experience, and to complement existing treatment [13, 14]. Two surveys examined medical conditions that healthcare professionals would consider treating with CAM therapies [8, 35]. The majority of GPs would consider recommending CAM to patients with musculoskeletal problems, pain, back problems, and for women’s health [8]. When treating pregnant women, physiotherapists used acupuncture for pregnancy-related complaints, including musculoskeletal pain, nausea, induction of labour, and breech presentation [35].

### Attitudes and views toward CAM

Out of the eight survey studies, healthcare professionals’ attitudes and views toward CAM were evaluated as a dichotomous outcome (either positive or negative toward CAM therapy use) [13] or in the form of statement agreement [8, 14, 15, 32]. In general, seven studies (five surveys, one focus group study, and one interview study) reported an overall positive attitude regarding use of CAM therapies [8, 9, 13–15, 32, 33]. Reasons in support of CAM use included the effectiveness of CAM and the view that CAM as a useful complement to conventional treatments [13]. On the other hand, perceived lack of evidence, lack of regulation, potential side effects, interference with other medications, and the financial cost for CAM represented the major concerns about CAM among GPs [13–15]. For Plunket nurses, despite positive perspectives on CAM, the organizational policy constraints and possible liability in engaging with CAM were the most significant barriers for CAM recommendation [9].

Surveys focusing on GPs showed that the majority of GPs insist on the need for an evidence base for CAM therapies, and encourage more scientific testing of CAM therapies before being used to complement conventional medicine [8, 14]. Notwithstanding this, findings of in-depth interviews with GPs acknowledged the importance of the patient-centred element that CAM therapies provide as being as important as the evidence-base [33]. For midwives, understanding and perception of CAM is closely linked to the philosophy, care perspectives, and professional goals of midwifery practice; over 70% of midwives perceived that CAM is an essential and traditional part of midwifery practice, and that the use of CAM enhances midwifery care [32].

Five studies [3, 9, 14, 15, 35] showed that on average 45.3% of healthcare professionals have concerns on the safety of CAM therapies. In particular, 87% of GPs and specialists were concerned about the safety of CAM use for oncology patients [15].

Two studies [12, 13] found no significant difference in the practicing patterns of CAM use by GPs by gender or age. Female, younger, and community doctors were more likely than their male, older, and hospital colleagues to refer patients to CAM practitioners [13, 15].

### Knowledge and education regarding CAM

Of the 11 studies included in this review, four [3, 8, 12, 13] reported healthcare professionals’ knowledge about CAM therapies. Knowledge about CAM was assessed by asking 1) whether healthcare professionals were familiar with or had heard of CAM therapies [8, 12], and 2) whether they knew local CAM practitioners [3, 12, 13]. In general, acupuncture is the most well-known CAM therapy among GPs [8, 12]. The mean percentage who knew local CAM practitioners was 84.7% with the highest rate of 94% [12]. A majority of GPs commented that they were unfamiliar with rongoā Māori and traditional Pacific Island medicine [3, 14]. Workshops, reading the literature, and discussions with colleagues were the major sources of obtaining CAM knowledge [32]. GPs expressed the need for ongoing peer support as well as access to reliable and unbiased CAM knowledge resources [33]. Approximately 38% of doctors felt comfortable discussing CAM use with patients [14, 15], and over 90% asked patients about their CAM use during consultation [3, 15], although only 40.6% initiated the discussion [32].

Four studies reported on issues associated with professionalism [3, 8, 33, 34]. Approximately 85% of GPs would like to see better regulation of CAM practitioners and products [3, 8]. It was reported that professional issues are important if GPs were considering any form of networking with CAM practitioners [33]. Community pharmacists recognized the current inadequacy of CAM regulations in New Zealand, and supported a national regulatory framework for both CAM practitioner and products to address concerns regarding CAM product quality, inappropriate health claims, and supporting evidence; there were mixed views as to whether regulation should be mandatory or self-regulation [34].

Findings indicated that approximately one quarter of GPs have received formal training in CAM [3, 12–14, 33], while 44.4% of physiotherapists received (postgraduate) training in acupuncture [35]. Surprisingly, it would appear that a number of GPs practised CAM therapies without training (i.e., acupuncture, chiropractic, and hypnosis) [12]. Interest in CAM among New Zealand healthcare professionals is high: up to 58% of GPs wished to receive further education on CAM [3, 8, 12, 14, 15]; moreover, a focus group study reported that all Plunket nurses wished to increase their knowledge about CAM modalities [9]. The two therapies in which GPs were the most interested in receiving training were acupuncture, and herbal medicine [14]. Interest in training declined in GPs aged over 50 years [12]. There is evidence that many healthcare professionals identify gaining knowledge about CAM as an important professional issue, and up to 66.7% GPs support the idea that an overview of CAM should be included into initial medical education [3, 14].

### Difference in CAM practice, attitudes and knowledge between healthcare professionals

Due to the wide variation in CAM therapies covered across the included studies, head-to-head comparisons between different categories of healthcare professionals could not be performed. Descriptive summaries from each discipline (when data were available) are reported above.

### Changes in CAM practice, attitudes, and knowledge over time

**Fig. 2:**
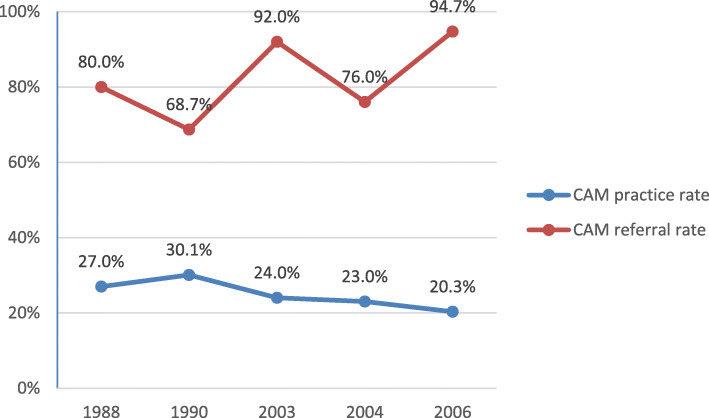
Changes over time in GPs’ CAM practice and referral patterns

Due to the limited number of studies on healthcare professionals other than GPs, only changes in GPs’ behaviours over time (based on data from five studies [3, 8, 12–14]) were evaluated (Fig. 2). In general, practice of CAM has slightly decreased since 1990, but the number of GPs trained in CAM has increased over time. Referral patterns for CAM fluctuated. Acupuncture is the most useful CAM therapy from GPs’ perspective, and is also the most commonly practiced, and referred CAM therapy by GPs over time (Tables 3, 4 and 5).

**Table 3:** Changes over time in GPs’ opinions on the usefulness of specific CAM therapies

Year	1st	2nd	3rd
1990 [13]	Acupuncture	Massage	Hypnosis
2003 [3]	Acupuncture	Chiropractic	Hypnosis
2006 [14]	Acupuncture	Chiropractic	Osteopathy

**Table 4:** Changes over time in GPs’ practice patterns of specific CAM therapies

Year	1st	2nd	3rd
1988 [12]	Acupuncture	Chiropractic	Hypnosis
1990 [13]	Acupuncture	Osteopathy	Homeopathy
2006 [14]	Acupuncture	Herbal medicines	Homeopathy

**Table 5:** Changes over time in GPs’ referral patterns for specific CAM therapies

Year	1st	2nd	3rd
1988 [12]	Acupuncture	Chiropractic	Hypnosis
1990 [13]	Acupuncture	Osteopathy	Massage
2004 [8]	Acupuncture	Homeopathy	Osteopathy
2006 [14]	Acupuncture	Chiropractic	Osteopathy

## Discussion

This is the first review that investigated New Zealand healthcare professionals’ practice of, attitudes toward, and knowledge about CAM. Review of the literature found 11 studies conducted with a total of 2060 healthcare professionals including GPs, Plunket nurses, midwives, pharmacists, physiotherapists, and specialists. Findings indicated that CAM use is common among New Zealand healthcare professionals: around 25% of GPs practise CAM, and 82.3% refer patients to CAM practitioners; when treating pregnant women, 48.4% of physiotherapists practise acupuncture, and 37.3% of midwives recommend CAM. GPs believe that acupuncture is the most useful CAM modality, and most commonly practiced and referred patients to acupuncture. New Zealand healthcare professionals are overall positive regarding CAM use, but have concerns on the scientific evidence, regulation, safety, financial costs of CAM, and encourage an evidence-based CAM practice and a stronger CAM regulation. Up to 58% of GPs and Plunket nurses wanted to receive further education on CAM, and up to 66.7% GPs favour the idea CAM should be included in medical curriculums.

A high uptake of CAM among New Zealand healthcare professionals was identified by this review. The estimates of CAM practice and referral rates are comparable to those oversea figures: 20.6% of United Kingdom (UK) physicians used CAM [37] and 84% of Australian rehabilitation physicians referred patients to CAM practitioners [38]. The overall positive attitude of CAM among New Zealand healthcare professionals is similar to those seen in as surveys of physicians in the UK [39, 40], with many healthcare professionals considering CAM as natural and effective, and regard the use of CAM as a complement to conventional therapy. The growing awareness of the importance of holistic health, where different aspects of health (mind, body, spirit, and lifestyle) combine to provide total wellbeing, as well as the need for culturally sensitive health services, are important factors that support consideration of the use of CAM [12, 15].

Our review showed the differences in referral patterns with respect to age, gender, and location of practice: the doctors who recommended CAM to a higher percentage of patients are more likely to be younger, female, and doctors who practice in the community (as opposed to a hospital). These results reflect similar patterns of clinical behaviours of healthcare professionals in the United States (US) and Canada [10]. Although these findings are interesting, as yet, there does not seem to be evidence to explain as to why this may be so. This perhaps indicates a changing culture, towards less conservative attitudes. This may be because of an increased exposure to CAM, and a higher likelihood of personal CAM use by younger physicians [10]. It may be because community doctors deal with a variety of health conditions, have more opportunities to address patients’ total wellbeing, and tend to be more familiar with patients [15].

Alongside the growing interest and use of CAM among New Zealand healthcare professionals have come a rising concern over the risks of using CAM. This is especially the case given that many healthcare professionals have received limited (formal) training on CAM, and some practise CAM without training. This finding is consistent with the results of similar UK studies: a sizable proportion of physicians in the UK employed CAM, yet many have not received any training in CAM [37]. While it is argued that CAM practitioners should have the ‘standard’ training and qualifications for CAM practice, this would also apply to conventional healthcare professionals who practise CAM. Specific training needs to be made available to enable them to meet expected standards of practice. In line with the findings of this review, in recent years there have been calls among healthcare professionals overseas (e.g., US and Germany) for further CAM education, and for the introduction of CAM courses in medical education institutions [41, 42]. National initiatives to integrate CAM elements in undergraduate medical education has been launched in a number of ‘developed’ countries including UK, US, Canada, Germany, and Australia [43]. Currently, CAM has been largely ignored in the undergraduate medical curriculum in New Zealand [9]; postgraduate courses on CAM are limited in universities, with postgraduate acupuncture courses for healthcare practitioners only available in University of Otago [44] and Auckland University of Technology [45]. GPs have consistently identified their limited knowledge about rongoā Māori and traditional Pacific Island medicine, but relevant education courses are still lacking. Despite the limited education resources, a number of medical professional and regulatory organizations in New Zealand, such as the *Medical Council of New Zealand* [46], the *New Zealand College of Midwives* [47], and *New Zealand Nurses Organisation* [48], have issued position statements endorsing the care standards of CAM practice, education, and knowledge. In responding to the increasing popularity and requirement for CAM, diverse training offerings including undergraduate, graduate clinical, and continuing education are warranted, which will inform healthcare professionals with up-to-date and scientific information.

To meet the challenges of managing CAM in New Zealand clinical settings, there is a need to encourage greater recognition, communication, and cooperation between conventional and CAM practitioners. Firstly, developing good inter-professional relationships will depend on an established common ground by way of adequate medical training and qualifications. Apart from the specific CAM training initiatives for conventional healthcare professionals (mentioned above), more formal medical training for CAM practitioners is essential. Such training could help reassure conventional healthcare professionals that CAM practitioners are able to communicate using a common medical language, and that serious conditions will not be unrecognized and inappropriately diagnosed, which are the concerns from GPs and pharmacists identified in the included two interview studies [33, 34].

Secondly, enhancing CAM credibility and acceptability to conventional healthcare professional will depend on established regulatory standards. As noted in the included studies, the relative hesitancy of healthcare professionals is partly due to the current absence of regulation for CAM, and the apparent lack of accountability [33, 34]. Although there have been mixed views to the regulation form (mandatory or self-regulation), healthcare professionals support proposals for national regulations for CAM. Chiropractic and osteopathy are the only two CAM therapies that have been regulated under the *HPCA Act 2003* [18]. It is acknowledged that full regulation of CAM in line with requirement of conventional medicines is problematic due to the wide variety of therapies under the CAM umbrella, and the relevant negative impacts on CAM market [34]. It would be more appropriate and feasible to introduce more regulations for the ones that are widely accepted and used by New Zealand healthcare professionals, for example, acupuncture. Chinese Medicine (which includes acupuncture) has being considered for regulation over the past five years. However, progress has been relatively slow with political and industry opposition, and no such regulatory has yet been made (unpublished: internal updates to members of *New Zealand Acupuncture Standards Authority*).

Thirdly, more efforts and supports from the government and private sector are needed to enhance the evidence base of CAM therapies. Many of the healthcare professionals in our review highlighted the lack of scientific evidence as a major concern about CAM, and the fact that their advice to patients on CAM is based on evidence. CAM research has been largely lacking in New Zealand: taking acupuncture for instance, five clinical trial studies on acupuncture were conducted in New Zealand since 2000 (identified by the *Australian New Zealand Clinical Trials Registry* [*ANZCTR*] [49]), compared to 75 in Australia (identified by *ANZCTR* [50]), and 70 in the UK (identified by the *International Standard Randomised Controlled Trial Number* [*ISRCTN*] [51]). It is frustrating, however, not surprising, to note the cycle of ‘lack of funding – lack of research – absence of evidence’ on CAM in New Zealand. It may not be pragmatic to set CAM as a priority for New Zealand funding bodies in the short term, but establishing open and non-judgemental perspectives on CAM research is encouraged. While CAM practitioners are supportive of evidence-based practice, absence of available evidence, industry support, and skills are perceived as the main barriers to evidence-based practice uptake [52]. Given that the research environment of CAM in New Zealand, in order to form a coordinated research activity and ensure appropriate scientific research quality, a CAM-based practice-based research network (which affiliates academic institutions and clinical practices to ask and answer community-based healthcare questions and translate research findings into practice) [53] may be a potential solution to better address contemporary CAM research needs in New Zealand.

Previous studies reported that physicians are more negative compared to other healthcare professionals, and nurses are found to be more accepted to CAM [9, 10]. This review was unable to compare the differences in clinical behaviours and attitudes between healthcare professionals due to a lack of studies of the healthcare professionals other than GPs. For the included studies on allied healthcare professionals (nurses, midwives, pharmacists and physiotherapists), the investigated CAM modalities, research questions, and methodology approaches were heterogeneous. Future research is needed to compare the practice of, attitudes toward, and knowledge about CAM amongst different categories of healthcare professionals, and advance the understanding of values and roles CAM in New Zealand healthcare systems. To enable direct comparisons, measurements of attitudes and knowledge require the use of reliable instruments, which are yet to be developed.

This is the first work that synthesises New Zealand healthcare professionals’ practice of, attitudes toward, and knowledge about CAM. This review summarized the results of existing quantitative and qualitative studies. To enhance its internal and external validity, this review strictly followed the methodology framework for integrative reviews, as well as the recommendations of the PRISMA statements. Nine of the 11 included studies were of moderate to high quality, thus enhancing the reliability of the review findings. This notwithstanding, there are limitations to be considered when interpreting the results. The definition of CAM used in the included studies raises difficulties in a review of this sort as there is a lack of consensus on what is meant by CAM. In addition, the low response rates in some surveys may introduce bias into research results that those healthcare professionals with strong views for or against CAM, may be overrepresented.

## Conclusions

Based upon the included 11 studies (two of ‘high-quality’, seven of ‘moderate-quality’, and two of ‘low-quality’), it was demonstrated that in order to better manage CAM in New Zealand clinical settings, there is a need to invest in CAM research and education, and enhance CAM regulation. This review is a first step in developing an evidence base to offer insights for further development of effective CAM policies regarding safety, efficacy, regulation and integration in New Zealand.

### Supplementary Information

**Additional file 1.** Risk of bias assessment of eight surveys by using the Hoy 2012 tool.

**Additional file 2.** Risk of bias assessment of three qualitative studies by using the CASP checklist.

## Data Availability

All data generated or analysed during this study are included in this published article and its supplementary information files.

## Abbreviations

CAM: Complementary and alternative medicine
GP: General practitioner
MACCAH: Ministerial Advisory Committee on Complementary and Alternative Health
ACC: Accident Compensation Corporation
PRISMA: Preferred Reporting Items for Systematic Reviews and Meta-Analyses
HPCA: Health Practitioners Competence Assurance
CASP: Critical Appraisal Skills Programme
UK: United Kingdom
US: United States
ANZCTR: Australian New Zealand Clinical Trials Registry
ISRCTN: International Standard Randomised Controlled Trial Number
