# Supplementary material for: Complementary and alternative medicine - practice, attitudes, and knowledge among healthcare professionals in New Zealand: an integrative review
Source: BMC Complement Med Ther. 2021 Feb 13;21:63. doi: 10.1186/s12906-021-03235-z (PMC7882070; doi:10.1186/s12906-021-03235-z)
Supplement: Supplementary file 1 — Additional file 1. Risk of bias assessment of eight surveys by using the Hoy 2012 tool. [file 12906_2021_3235_MOESM1_ESM.docx]

**Additional file 1:** Risk of bias assessment of eight surveys by using the *Hoy 2012 tool*

| **Author (Year)** | **1. Representation** | **2. Sampling** | **3. Random selection** | **4. Non response bias** | **5. Data collection** | **6. Case Definition** | **7. Reliability and validity of study tool** | **8. Method of data collection** | **9. Prevalence period** | **10. Numerator and denominator** | **overall risk of bias** |
| --- | --- | --- | --- | --- | --- | --- | --- | --- | --- | --- | --- |
| Hadley (1988) | No | Yes | Yes | Yes | Yes | Yes | No | Yes | No | Yes | Moderate |
| Marshall (1990) | No | Yes | Yes | No | Yes | No | No | Yes | No | Yes | High |
| Taylor (2003) | No | Yes | Yes | Yes | Yes | No | No | Yes | No | Yes | Moderate |
| Lawler (2004) | No | Yes | Yes | No | Yes | Yes | No | Yes | No | Yes | Moderate |
| Poynton (2006) | Yes | Yes | Yes | No | Yes | Yes | No | Yes | No | Yes | Moderate |
| Harding (2009) | Yes | Yes | Yes | No | Yes | No | No | Yes | No | Yes | Moderate |
| Bocock (2011) | No | Yes | Yes | No | Yes | No | No | Yes | No | Yes | High |
| McDowell (2019) | Yes | Yes | Yes | No | Yes | Yes | No | Yes | No | Yes | Moderate |
| Risk of bias assessment tool (Hoy 2012): Yes (low risk); No (high risk)  1. Representation: Was the study’s target population a close representation of the national population?  2. Sampling: Was the sampling frame a true or close representation of the target population?  3. Random selection: Was some form of random selection used to select the sample, OR, was a census undertaken?  4. Non-response bias: Was the likelihood of non-response bias minimal?  5. Data collection: Were data collected directly from the subjects?  6. Case definition: Was an acceptable case definition used in the study?  7. Reliability and validity of study tool: Was the study instrument that measured the parameter of interest shown to have reliability and validity?  8. Data collection: Was the same mode of data collection used for all subjects?  9. Prevalence period: Was the length of the prevalence period for the parameter of interest appropriate?  10. Numerators and denominators: Were the numerator(s) and denominator(s) for the parameter of interest appropriate? | | | | | | | | | | | |
