# Supplementary material for: Complementary and alternative medicine - practice, attitudes, and knowledge among healthcare professionals in New Zealand: an integrative review
Source: BMC Complement Med Ther. 2021 Feb 13;21:63. doi: 10.1186/s12906-021-03235-z (PMC7882070; doi:10.1186/s12906-021-03235-z)
Supplement: Supplementary file 2 — Additional file 2. Risk of bias assessment of three qualitative studies by using the CASP checklist. [file 12906_2021_3235_MOESM2_ESM.docx]

**Additional file 2:** Risk of bias assessment of three qualitative studies by using the *CASP checklist*

| **Author (Year)** | **1. Statement of research aims** | **2. Appropriate qualitative methodology** | **3. Appropriate research design** | **4. Appropriate recruitment strategy** | **5. Data collection justified** | **6. Relationship between researcher and participants considered** | **7. Ethical issues considered** | **8. Rigorous data analysis** | **9. Clear statement of findings** | **10. Value of research** | **Overall study quality** |
| --- | --- | --- | --- | --- | --- | --- | --- | --- | --- | --- | --- |
| Upsdell (2011) | Yes | Yes | Yes | Yes | Yes | No | Yes | No | No | No | Moderate |
| Lo (2012) | Yes | Yes | Yes | Yes | Yes | Yes | Yes | Yes | Yes | Yes | High |
| Barnes (2018) | Yes | Yes | Yes | Yes | Yes | Yes | Yes | Yes | Yes | Yes | High |
| Risk of bias assessment tool (CASP 2018):  1. Was there a clear statement of the aims of the research?  2. Is a qualitative methodology appropriate?  3. Was the research design appropriate to address the aims of the research?  4. Was the recruitment strategy appropriate to the aims of the research?  5. Was the data collected in a way that addressed the research issue?  6. Has the relationship between researcher and participants been adequately considered?  7. Have ethical issues been taken into consideration?  8. Was the data analysis sufficiently rigorous?  9. Is there a clear statement of findings?  10. How valuable is the research? | | | | | | | | | | | |
